# Machine Learning-Based Prediction of Coronary Artery Disease Using Clinical and Behavioral Data: A Comparative Study

**DOI:** 10.3390/diagnostics16020318

**Published:** 2026-01-19

**Authors:** Abdulkadir Çakmak, Gülşah Akyilmaz, Aybike Gizem Köse, Gökhan Keskin, Levent Uğur

**Affiliations:** 1 Department of Cardiology, Faculty of Medicine, Amasya University, Amasya 05200, Türkiye; cakmaka6@gmail.com (A.Ç.);; 2 Department of Nutrition and Diet, Private Kolmed Hospital, Amasya 05100, Türkiye; gulsahyildizakyilmaz@gmail.com; 3 Department of Nutrition and Dietetics, Faculty of Health Sciences, Amasya University, Amasya 05200, Türkiye; aybike.kayacan@amasya.edu.tr; 4 Department of Mechanical Engineering, Faculty of Engineering, Amasya University, Amasya 05100, Türkiye

**Keywords:** coronary artery disease, machine learning, diagnostic model, clinical data, psychosocial factors, cardiovascular risk

## Abstract

**Background and Objectives:** Coronary artery disease (CAD) is a leading cause of morbidity and mortality worldwide. An early and accurate diagnosis is essential for effective clinical management and risk stratification. Recent advances in machine learning (ML) have provided opportunities to enhance the diagnostic performance by integrating multidimensional patient data. This study aimed to develop and compare several supervised ML algorithms for early CAD diagnosis using demographic, anthropometric, biochemical, and psychosocial parameters. **Materials and Methods:** A total of 300 adult patients (165 CAD-positive and 135 controls) were retrospectively analyzed using a dataset comprising 21 biochemical markers, body composition metrics, and self-reported eating behavior scores. Six ML algorithms, k-nearest neighbors (k-NNs), support vector machines (SVMs), artificial neural networks (ANNs), logistic regression (LR), naïve Bayes (NB), and decision trees (DTs), were trained and evaluated using 10-fold cross-validation. Model performance was assessed based on accuracy, sensitivity, false-negative rate, and area under the Receiver Operating Characteristic (ROC) curve (AUC). **Results:** The k-NN model achieved the highest performance, with 98.33% accuracy and an AUC of 0.99, followed by SVM (96.67%, AUC = 0.95) and ANN (95.33%, AUC = 0.98). Patients with CAD exhibited significantly higher levels of glucose, triglycerides (TGs), LDL cholesterol (LDL-C), and abdominal obesity, while vitamin B12 levels were lower (*p* < 0.001). Although emotional and mindful eating scores differed significantly between the groups, their contribution to model performance was limited. **Conclusions:** Machine learning models, particularly k-NN, SVM, and ANN, have demonstrated high accuracy in distinguishing CAD patients from healthy controls when applied to a diverse set of clinical and behavioral variables. This study highlights the potential of integrating psychosocial and clinical data to enhance CAD prediction models beyond traditional biomarkers.

## 1. Introduction

Coronary artery disease (CAD) is a major public health problem worldwide, with high mortality and morbidity rates. Both clinical and epidemiological data indicate that CAD poses a significant healthcare burden not only in developed countries, but also in low- and middle-income nations [1]. This underscores the critical need for the development of early diagnostic and risk assessment strategies, allowing timely interventions that can alter the course of the disease [2].

Traditional diagnostic methods are limited to clinical evaluation and noninvasive imaging techniques. Therefore, the development of novel biomarkers and predictive models for early detection of CAD is of great importance. In particular, the combination of body composition analysis and metabolic parameters offers a promising approach for predicting disease prognosis [3]. In this context, the integrated assessment of multiple parameters may allow for more accurate and personalized identification of CAD.

Conventional clinical approaches for assessing CAD risk often rely on invasive methods, which may pose additional risks to patients. To overcome this limitation, a growing number of models have recently been developed that utilize non-invasive data, such as individuals’ anthropometric characteristics (e.g., waist circumference, body mass index (BMI), muscle mass) and biochemical indicators (e.g., glucose, glycated hemoglobin (HbA1c), and lipid profile) [4]. These indicators are considered foundational tools for the quantitative evaluation of cardiovascular risk and individualization of treatment strategies [2]. Furthermore, large-scale cohort studies that collect such data enable more sophisticated and comprehensive analyses of risk modeling [5].

In addition, the potential impact of psychosocial eating behaviors on CAD risk has attracted increasing attention in recent years. Behavioral factors such as emotional eating and eating awareness influence individuals’ dietary habits, and consequently, their metabolic profiles [6]. Assessing the effects of psychosocial conditions and lifestyle factors on cardiovascular risk may go beyond conventional risk scores, offering a more holistic approach to risk assessment [2]. In particular, combining subjective self-reported data with objective biochemical and anthropometric markers may more accurately reflect individuals’ risk profiles [4].

In recent years, machine learning (ML) has emerged as a widely used method in medical research. These techniques offer significant advantages in identifying hidden patterns within large and complex datasets and in predicting disease diagnosis and prognosis [7,8,9,10]. ML algorithms integrate conventional clinical data with biochemical, anthropometric, and behavioral indicators, enabling a more accurate prediction of CAD risk [11]. Models developed using this approach have demonstrated improved accuracy in patient-specific risk predictions and have become essential components of clinical decision-support systems [1]. Moreover, the data processing techniques and model optimization strategies employed allow meaningful insights to be derived from large datasets [4]. In particular, supervised learning algorithms provide high accuracy in the classification of clinical data and serve as valuable tools for assisting physicians in decision making. In the present study, ML models were used to investigate the relationship between CAD and demographic, anthropometric, and biochemical markers.

However, although many studies have focused solely on predicting the presence of CAD, few have developed classification models based on a comprehensive evaluation of clinical and biochemical parameters associated with the disease. However, CAD can present a heterogeneous clinical course among individuals, and there is an increasing need for personalized risk stratification for disease management. Stratifying patients according to their demographic, anthropometric, and biochemical characteristics may allow more effective planning of diagnostic and therapeutic processes. Furthermore, this study investigates how demographic (age, sex), anthropometric (BMI, waist circumference, body fat percentage), and biochemical (glucose, HbA1c, lipid profile) parameters differ between individuals with and without a confirmed CAD diagnosis. Machine learning algorithms, including k-nearest neighbors (k-NNs), support vector machines (SVMs), and artificial neural networks (ANNs), were applied to identify the most influential predictors among these variables and to determine their relative contributions to model performance [12,13]. Integrating heterogeneous clinical data sources—such as anthropometric and biochemical markers—has been shown to enhance classification accuracy and improve personalized CAD risk stratification compared with traditional risk factors alone [12,14]. Such models can support early detection and individualized management by providing decision-support insights that go beyond conventional scoring systems [14]. However, challenges remain in harmonizing heterogeneous data formats, managing missing values, and ensuring reproducibility across institutions—a key focus of this research. Accordingly, the study aimed to develop ML models that classify CAD presence while exploring clinical profile differences across demographic, anthropometric, and biochemical dimensions. This approach is expected to make significant contributions to the development of personalized decision support systems and provision of proactive healthcare services.

### Related Work and Summary of Literature

Numerous studies have applied ML techniques for the diagnosis of CAD using various combinations of clinical, demographic, and imaging data. However, relatively few studies have integrated the demographic, biochemical, anthropometric, and psychosocial dimensions simultaneously within a multivariate framework.

Table 1 provides a comparative summary of recent and relevant studies that applied ML algorithms to CAD prediction. This literature synthesis highlights the diversity of methodological approaches and data types used across studies and sets the foundation for the integrated multivariate strategy employed in our research.

## 2. Materials and Methods

This study was conducted to develop and evaluate machine learning models for the prediction of coronary artery disease (CAD) using demographic, anthropometric, biochemical, and psychosocial data obtained from clinical records. In total, the dataset included 21 biochemical parameters (glucose, HbA1c, triglycerides, HDL-C, LDL-C, total cholesterol, liver enzymes, thyroid hormones, vitamin B12, ferritin, hemoglobin, etc.), six anthropometric measurements (height, weight, waist and hip circumference, body-fat percentage, and muscle mass), and two psychosocial scales (Emotional Eating Scale and Mindful Eating Questionnaire). These variables were selected to provide a comprehensive clinical and behavioral profile of each participant.

### 2.1. Patient Characteristics

This retrospective study included data from adult individuals aged ≥ 18 years who had previously presented to the Cardiology Outpatient Clinic of Amasya University Faculty of Medicine between 11 February 2025 and 12 July 2025. After obtaining ethical approval, relevant clinical records, including anthropometric measurements, body composition analyses, and blood biochemical parameters, were extracted from the hospital database. In this study, a confirmed diagnosis of CAD was defined as the presence of angiographically verified coronary atherosclerosis or evidence of myocardial ischaemia demonstrated by stress echocardiography, myocardial perfusion scintigraphy (MPS), or computed tomography angiography (CTA). Standard resting electrocardiography (ECG) and echocardiography (Echo) were used for baseline cardiac evaluation, while stress testing was performed in patients with ambiguous or borderline findings. Individuals with previous myocardial revascularization (percutaneous coronary intervention or coronary artery bypass grafting) were excluded to avoid bias in diagnostic labeling. The study protocol was approved by the Amasya University Ethics Committee (decision number: 2024/177). Owing to the retrospective nature of the study, the requirement for obtaining informed consent from patients was waived by the Amasya University Rectorate Non-Interventional Clinical Research Ethics Committee.

The diagnosis of CAD was confirmed based on clinical evaluation findings, noninvasive imaging methods (such as ECG, Echo, CTA, and MPS), and when necessary, conventional coronary angiography (CAG) examinations.

Among these diagnostic modalities, CTA and MPS contributed most frequently to the confirmation of CAD, followed by Echo and resting ECG analysis. The retrospective dataset contained varying completeness across modalities; approximately 15% of the initial records lacked full biochemical data and 8% lacked imaging documentation, which were excluded prior to analysis. All remaining records were cross-checked by two cardiologists to ensure data integrity and reduce misclassification bias arising from retrospective chart review.

Inclusion criteria were adult individuals with a complete dataset who had either a confirmed or excluded diagnosis of CAD. Patients with serious systemic illnesses that could significantly affect biochemical or anthropometric parameters (e.g., advanced liver disease, chronic renal failure, active malignancy), those who experienced acute coronary syndrome (ACS) or another acute cardiovascular event during data collection, and pregnant or breastfeeding women were excluded from the study.

The records of 2500 patients were reviewed, and after applying strict inclusion and exclusion criteria, the study included data from 300 patients who met all eligibility requirements. Among them, 165 patients were diagnosed with CAD, while the remaining 135 were assigned to the control group. The overall sex distribution in the study population was relatively balanced, with 160 males (53.3%) and 140 females (46.7%). Within the CAD group, 92 patients were male (55.8%) and 73 were female (44.2%), whereas the control group consisted of 68 males (50.4%) and 67 females (49.6%).

Although the initial database comprised 2500 patient records, only 300 cases fulfilled the predefined quality and completeness criteria after excluding incomplete or confounding data (e.g., missing anthropometric parameters, systemic diseases, acute coronary syndromes, or previous revascularization). This reduction reflects deliberate data curation and quality control rather than data loss or selection bias. Comparable sample sizes have been reported in other exploratory ML-based CAD studies supporting the methodological consistency of our approach [16,20,24,26].

The demographic and clinical characteristics of the study population are detailed in Table 2, including the distribution of age, sex, anthropometric measurements, and blood biochemistry parameters.

**Figure 1:**
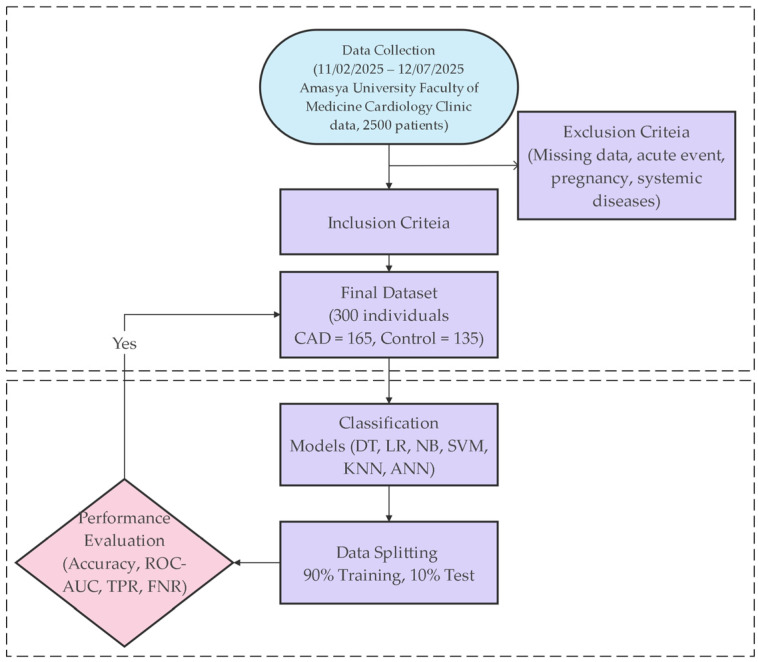
Workflow Diagram.

The methodological workflow employed in this study, from data collection to model evaluation, is illustrated in Figure 1. This flowchart summarizes all steps, including the inclusion criteria, modeling processes, and performance assessments.

### 2.2. Body Measurements

Various anthropometric data related to body measurements were collected from the participants in this study. Measurements included height, weight, waist circumference, hip circumference, body fat percentage, and muscle mass. The obtained values were compared between the control individuals and patients diagnosed with CAD, and statistical analyses were conducted. Table 3 presents the relevant findings. Anthropometric profiles showed pronounced differences between CAD and control groups across all parameters, with waist circumference and body fat percentage demonstrating the strongest predictive value. Excluding participants with systemic diseases (liver disease, renal failure, malignancy) or acute coronary syndrome (ACS) ensured a homogeneous and stable dataset. From the initial 2500 patient records, 2200 (88%) were excluded due to missing or incomplete entries, yielding a final analytical sample of 300 patients.

The analyses revealed statistically significant differences in all body measurements between individuals with and without CAD (*p* < 0.001). Notably, differences in abdominal obesity indicators, such as waist and hip circumferences, were particularly prominent. These findings support the potential role of abdominal adiposity in the development of CAD.

### 2.3. Laboratory Parameters

All biochemical and hormonal parameters assessed in this study are routinely measured in the clinical evaluation of patients with suspected or established coronary artery disease, as recommended by major cardiovascular guidelines [27,28]. In this study, various biochemical and hormonal data were collected from the participants. These included glucose, HbA1c, triglycerides (TGs), high-density lipoprotein cholesterol (HDL-C), low-density lipoprotein cholesterol (LDL-C), non-high-density lipoprotein cholesterol (non-HDL-C), aspartate aminotransferase (AST), alanine aminotransferase (ALT), alkaline phosphatase (ALP), gamma-glutamyl transferase (GGT), free triiodothyronine (FT3), free thyroxine (FT4), thyroid-stimulating hormone (TSH), and clinically relevant indicators, such as vitamin B12, ferritin, and hemoglobin (Hgb). In addition, metabolic risk indices, such as the triglyceride-glucose (TyG) index, Atherogenic Index of Plasma (AIP), and LDL-C/HDL-C ratio, were calculated. The collected data were compared between individuals with and without CAD, and the results are summarized in Table 4.

In this retrospective cohort, biochemical variables such as glucose, HbA1c, triglycerides, and LDL-C, together with anthropometric measures including waist circumference and BMI, were most strongly associated with confirmed CAD (all *p* < 0.001). Non-invasive imaging modalities such as coronary CTA and myocardial perfusion scintigraphy (MPS) were the most frequent methods contributing to CAD confirmation, whereas resting ECG and echocardiography served as baseline assessments.

Statistical analyses revealed that individuals with CAD had significantly higher levels of several biochemical parameters, particularly glucose, HbA1c, LDL-C, TG, and TC, than control individuals (*p* < 0.001). In contrast, vitamin B12 levels were significantly lower in the CAD group. These findings highlight an association between CAD and metabolic dysregulation.

### 2.4. Emotional Eating and Eating Awareness Data

In this study, data from the Emotional Eating Scale (EES) and Mindful Eating Questionnaire (MEQ) were analyzed to evaluate the psychosocial aspects of participants’ eating behaviors. Emotional eating reflects the tendency of individuals to eat in response to emotional states such as stress, anger, or anxiety, while mindful eating measures an individual’s sensitivity to physical hunger and satiety cues as well as their ability to maintain awareness during eating behavior [7].

The EES was significantly higher in the CAD group (mean = 3.18, SD = 1.11) than in the control group (mean = 3.94, SD = 0.36; *p* < 0.001). Similarly, the MEQ was lower in the CAD group (mean = 3.39, SD = 0.73) than in the controls (mean = 3.66, SD = 0.18; *p* < 0.001).

To ensure the appropriateness of subsequent statistical tests, the normality of the score distributions was assessed using the Kolmogorov–Smirnov test. The results confirmed that both variables were normally distributed, thereby supporting the use of parametric statistical methods for further analyses. Pregnancy-related physiological changes and hormonal fluctuations were among the exclusion criteria, as they could affect anthropometric and biochemical markers. The observed higher Emotional Eating Scale (EES) and lower Mindful Eating Questionnaire (MEQ) scores among CAD patients suggest that behavioral tendencies—such as stress-related eating—may contribute indirectly to metabolic dysregulation and elevated cardiovascular risk [14]. These findings support the integration of psychosocial variables into CAD prediction models to capture behavioral components of disease risk.

### 2.5. Statistical Analysis

All statistical analyses were performed using Statistical Package for the Social Sciences (SPSS) version 25.0 (IBM Corp., Armonk, NY, USA).

The Shapiro–Wilk test was used to assess the normality of continuous variables. Normally distributed variables are presented as means ± standard deviations, whereas categorical variables are expressed as numbers (*n*) and percentages (%).

For comparisons between two groups, an independent samples t-test was used for continuous variables and the chi-square test was applied for categorical variables. Differences were considered statistically significant at a *p*-value < 0.05.

Additionally, to identify variables with high discriminative power for CAD diagnosis, Receiver Operating Characteristic (ROC) curve analysis was conducted for significantly different variables. The Area Under the Curve (AUC) was calculated for each parameter to evaluate its ability to differentiate patients from control individuals.

To control for Type I errors arising from multiple comparisons among the 21 biochemical parameters, a Bonferroni correction was applied. The adjusted significance level was set at *p* < 0.0024 (0.05/21). Variables meeting this adjusted threshold were considered statistically significant.

### 2.6. Classification

The classification process in this study was based on demographic, anthropometric, and biochemical data obtained from adults who visited the Cardiology Outpatient Clinic of the Amasya University Faculty of Medicine. The primary objective of this study was to develop models that could accurately and reliably predict CAD based on patient characteristics. The dataset was collected between 11 February 2025 and 12 July 2025, and each individual was labeled as either CAD-positive or CAD-negative.

All available features were included in the classification models without feature selection. This approach aims to leverage all potential sources of information and avoid the exclusion of variables that might have clinical relevance. In total, 21 biochemical parameters, including age, sex, body measurements, and derived indices (such as TyG index, AIP, and LDL-C/HDL-C ratio), were used as input variables. This decision ensured that all demographic, anthropometric, and biochemical variables contributed to the predictive process, allowing assessment of each variable’s relative weight in CAD classification. While this approach may reduce interpretability, it preserves data richness and avoids premature exclusion of potentially informative predictors [12].

The classification models were built using supervised learning algorithms. Several ML techniques were employed and compared, including Decision Trees (DTs), Support Vector Machines (SVMs), Naive Bayes (NB), k-nearest neighbors (k-NNs), Artificial Neural Networks (ANNs), and Logistic Regression (LR). Model performance was evaluated using various metrics: accuracy, sensitivity, specificity, F1-score, and AUC.

All continuous features were standardized using z-score normalization prior to model training to ensure comparability across biochemical and anthropometric parameters. Outliers were examined using the interquartile range (IQR) criterion, and extreme values were Winsorized to mitigate their influence while maintaining data integrity.

Model evaluation was performed using stratified 10-fold cross-validation, ensuring that class proportions (CAD vs. control) were preserved within each fold. In each iteration, 90% of the data were used for model training and 10% for validation, with a fixed random seed applied for reproducibility.

This approach enhanced the generalizability of the models across different data subsets, reduced the risk of overfitting, and yielded reliable performance metrics that can guide the development of clinically applicable decision support systems and contribute to the optimization of data-driven healthcare services.

#### 2.6.1. Decision Trees (DTs)

DT is a popular tool in classification algorithms due to its straightforward, flowchart-like structure. Internal nodes represent decision points, typically illustrated as rectangles, and leaf nodes, shown as ovals, indicate the outcomes. DTs are favored for their ease of interpretation and implementation compared to other classification methods. They are capable of handling both numerical and categorical data and are used in various applications, including machine learning, data mining, and pattern recognition [29,30].

#### 2.6.2. Support Vector Machines (SVMs)

Support Vector Machine (SVM) is a powerful and widely used supervised learning algorithm, introduced by Vladimir Vapnik, that aims to find the optimal hyperplane which best separates data points belonging to different classes. It is particularly effective in high-dimensional spaces and is frequently applied in biomedical informatics, image recognition, and text classification tasks due to its robustness and scalability [31,32]

#### 2.6.3. Naive Bayes (NB)

Naive Bayes (NB) is a probabilistic classification algorithm grounded in Bayes’ Theorem, which calculates the posterior probability of a class given a set of features. NB makes the “naive” assumption that all features are conditionally independent given the class label. Despite this simplification—often violated in real-world data—it remains highly effective, particularly in high-dimensional spaces. Its strengths include fast computation, minimal training data requirements, and suitability for both binary and multiclass classification tasks. NB is widely used in applications such as text classification, spam filtering, and medical diagnosis where probabilistic reasoning is beneficial [33,34]

#### 2.6.4. k-Nearest Neighbors (k-NNs)

The k-Nearest Neighbor (k-NN) algorithm is a simple yet powerful method for classification. It does not create a model during training. Instead, it stores all training data and makes predictions based on the nearest d value.

This non-parametric approach does not require a training phase, which makes it memory-intensive but straightforward. k-NN is particularly effective when decision boundaries are non-linear or irregular [35,36]

#### 2.6.5. Artificial Neural Networks (ANNs)

Artificial Neural Networks (ANNs) are a foundational technology in machine learning, mirroring the neural networks of the human brain. ANNs consist of interconnected nodes or neurons, where each connection represents a synaptic weight. The most common type of ANN is the feed-forward neural network, where information flows in one direction—from input nodes, through hidden layers (if any), to output nodes. A widely used version of this architecture is the Multilayer Perceptron (MLP), which is typically trained using the backpropagation algorithm. This method adjusts the weights of the network step by step to minimize the difference between predicted and actual outcomes [37,38].

#### 2.6.6. Logistic Regression (LR)

Logistic Regression (LR) is a foundational classification algorithm extensively used in biomedical research for binary outcome prediction.

The model is valued for its simplicity, interpretability, and computational efficiency. It provides direct insights into the influence of individual predictors via odds ratios, making it especially suitable for risk factor analysis in clinical settings. Despite its assumption of linearity, LR often serves as a strong baseline in classification tasks across medical diagnostics, social science research, and credit scoring applications [39,40]

### 2.7. Hyperparameter Tuning Details

Hyperparameter optimization was performed using MATLAB’s Classification Learner App (Version R2023b, MathWorks, Natick, MA, USA), which provides an interactive interface for training and comparing multiple machine learning classifiers. For each model, automated hyperparameter tuning was conducted using built-in options such as grid search and cross-validation.

During model tuning, 10-fold cross-validation was implemented within the MATLAB Classification Learner (CL) environment to minimize overfitting and ensure model generalizability. Within each fold, all preprocessing procedures—including feature normalization, scaling, and data partitioning—were independently applied to prevent information leakage between training and validation subsets. The app’s built-in optimization function performed a grid search across predefined parameter ranges (e.g., varying regularization strength for logistic regression, kernel type and box constraint for SVM, number of neighbors for k-NN, and number of hidden neurons for ANN).

Table 5 summarizes the hyperparameters explored and the best-performing values selected for each model. From a computational perspective, omitting feature selection increased training time but provided more comprehensive variable int

## 3. Results

**Figure 2:**
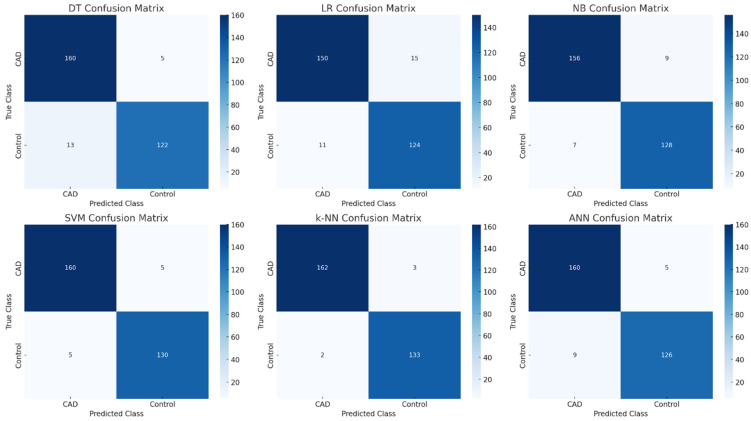
Confusion matrix heatmaps of all models.

In this study, various supervised ML algorithms were implemented to predict CAD, and their classification performances were compared. No feature selection was applied during the model development phase, and all demographic, biochemical, and anthropometric variables were included in the models. The classification performance results are visualized in the confusion matrix heatmaps presented in Figure 2. The findings from this comprehensive dataset demonstrate the significant role of data diversity and richness in enhancing model performance. Indeed, the use of all available features enabled the models to accurately distinguish between control individuals and those diagnosed with CAD. This clearly emphasizes the critical importance of multidimensional and multidisciplinary data utilization in the development of clinical decision support systems.

As illustrated in Figure 2, the k-NN model correctly classified 162 of 165 CAD cases and 133 of 135 control subjects, yielding a True Positive Rate (TPR) of 98.18% and a False Negative Rate (FNR) of 1.82% for the CAD class. Similarly, the SVM and ANN models achieved TPR values above 96%, with only 5 and 5 CAD cases misclassified, respectively. The Decision Tree model misclassified 13 controls and 5 CAD cases, whereas Naïve Bayes achieved a balanced performance, misclassifying 7 controls and 9 patients. These results demonstrate that k-NN, SVM, and ANN models minimized false negatives, which is critical in a diagnostic context [12,13].

In these matrices, the rows represent the actual class labels (CAD and control), whereas the columns represent the predicted class labels. The diagonal cells indicate correct classifications (true positives and true negatives), whereas the off-diagonal cells reflect misclassifications.

The k-NN model demonstrated the most balanced and accurate performance, correctly classifying 162 out of 165 CAD cases and 133 out of 135 control individuals. SVM and ANN have achieved high classification consistency, particularly in minimizing false negatives in CAD cases. In contrast, the DT model exhibited the lowest performance, misclassifying 13 control individuals and five CAD cases. These findings visually corroborate the numerical performance metrics presented in Table 6, emphasizing the superior diagnostic capability of models such as k-NN, SVM, and ANN for CAD prediction.

Among the tested models, the k-NN algorithm achieved the highest classification accuracy of 98.33%. It demonstrated excellent performance in both classes, with a True Positive Rate (TPR) and False Negative Rate (FNR) of 98.18% and 1.82% for the CAD class and 98.52% and 1.48% for the control class, respectively. The SVM model also performed competitively, achieving an overall accuracy of 96.67% with TPR/FNR values of 96.97%/3.03% for CAD and 96.30%/3.70% for the control. The ANN followed with 95.33% accuracy, attaining 96.97%/3.03% for CAD, and 93.33%/6.67% for the control.

The other models showed moderate but noteworthy performance. The NB model achieved 94.67% accuracy with a TPR/FNR of 94.55%/5.45% for CAD and 94.81%/5.19% for the control. The DT model achieved 94.00% accuracy, although there was a greater disparity between classes: 96.97%/3.03% for CAD and 90.37%/9.63% for the control. The LR model exhibited the lowest accuracy at 91.33%, with 90.91%/9.09% for CAD and 91.85%/8.15% for the control. Overall, k-NN, SVM, and ANN demonstrated superior performance across both classes, with k-NN being the most balanced and effective model. These findings are detailed in Table 6, which presents a class-wise breakdown of model sensitivity (TPR), miss rates (FNR), and total accuracy.

In summary, individuals with CAD showed significantly higher glucose, HbA1c, triglycerides, and LDL-C levels, as well as greater waist circumference and body-fat percentage compared with controls (*p* < 0.001). In contrast, vitamin B12 levels were markedly lower among CAD patients. These findings suggest a clear metabolic and anthropometric risk profile characteristic of CAD. Among the evaluated algorithms, k-NN achieved the highest classification accuracy (98.33%, AUC = 0.99), followed by SVM (96.67%) and ANN (95.33%). Feature-importance analysis highlighted glucose, LDL-C, and waist circumference as the most influential predictors distinguishing CAD-positive from control individuals, consistent with the pathophysiological link between metabolic dysregulation and atherosclerosis.

In this study, ROC curves were generated, and the AUC values were calculated for each machine learning algorithm to assess their discriminative power in diagnosing CAD. ROC curves illustrate the trade-off between the TPR (sensitivity) and False Positive Rate (FPR) across various classification thresholds, thereby providing insight into each model’s ability to distinguish between CAD and control cases. The AUC value serves as a comprehensive metric for evaluating the overall model performance.

According to the results, the k-NN algorithm achieved the highest AUC value of 0.99, indicating an excellent class separation capability. The ANN and SVM models also performed well, with AUC values of 0.98 and 0.95, respectively. The NB model achieved an AUC of 0.97, followed by LR with 0.92, and the DT model with 0.91. Although these latter models exhibited relatively lower AUC scores, their discriminative performance remained within the acceptable limits for clinical applications.

**Figure 3:**
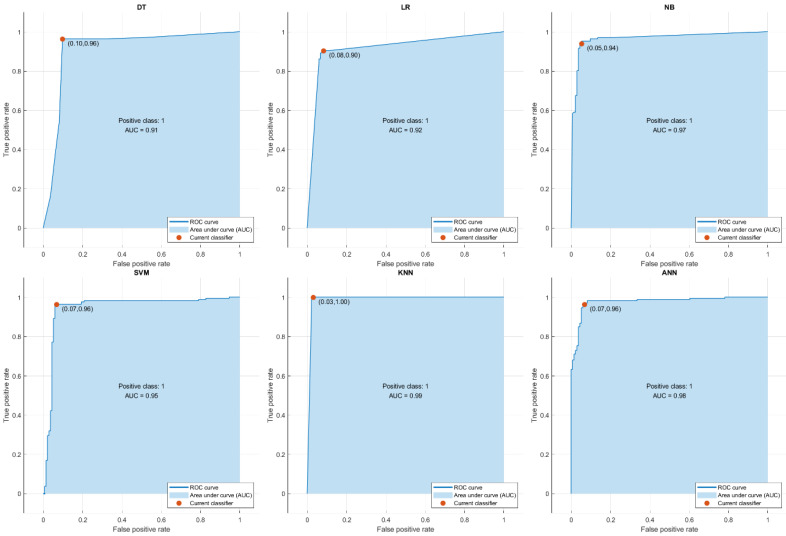
ROC curves and AUC values for the six different ML algorithms used in the study.

These findings confirm that models such as k-NN, ANN, and SVM are particularly effective in distinguishing between CAD-positive and control individuals. The AUC scores derived from the ROC analysis highlight not only the classification accuracy but also the potential integration of these models into clinical decision support systems (CDSS). The ROC curves and corresponding AUC values for each classifier are shown in Figure 3. ROC analysis further supported these findings. The AUC values were as follows: k-NN = 0.99, ANN = 0.98, SVM = 0.95, NB = 0.97, LR = 0.92, and DT = 0.91. According to these metrics, the three models demonstrating the strongest discriminative power were k-NN, ANN, and SVM, with AUC ≥ 0.95, consistent with recent evidence emphasizing the superiority of non-linear classifiers in CAD prediction [12,14].

Although LR and DT exhibited slightly lower AUC values (0.92 and 0.91, respectively), these remain within clinically acceptable limits for screening models, as AUC > 0.90 is often regarded as good diagnostic capability. The ROC curves illustrate how the True Positive Rate increases with an acceptable False Positive Rate, confirming the models’ applicability for clinical decision-support systems (CDSSs).

The performance metrics of the six machine learning algorithms evaluated in this study are presented in Table 7. The results were obtained using 10-fold cross-validation on the entire dataset (*n* = 300), where each data point served as validation exactly once and as part of the training data nine times. All preprocessing and hyperparameter tuning were performed within each fold to prevent data leakage.

In each iteration of the 10-fold cross-validation, approximately 90% of the dataset was used for training and 10% for validation, ensuring that every sample contributed once to testing and nine times to training. Preprocessing and hyperparameter tuning steps (normalization, scaling, and optimization) were repeated independently within each fold to prevent data leakage between subsets. This procedure mitigates overfitting and improves model generalizability by exposing algorithms to multiple data partitions rather than a single train–test split [14]. The overall accuracy difference between the best-performing model (k-NN, 98.33%) and the least-performing model (DT, 94%) was only 4.3%, confirming the robustness and stability of the 10-fold strategy.

Among the models, k-Nearest Neighbor (k-NN) achieved the highest performance with an accuracy of 98.33%, followed closely by Support Vector Machine (SVM) at 96.67% and Artificial Neural Network (ANN) at 95.00%. These results suggest that models capable of capturing non-linear relationships were more successful in distinguishing between CAD and non-CAD patients in this dataset.

Logistic Regression (LR) also performed strongly with an accuracy of 91.67%, demonstrating that even linear models can be effective when the data is informative and well-preprocessed. Naive Bayes (NB) and Decision Tree (DT) achieved accuracies of 88.33% and 86.00%, respectively, with DT performing the lowest among the six models.

The evaluation metrics—accuracy, precision, recall, F1-score, and AUC—were consistent with expected patterns, confirming the relative strength of non-linear models in complex classification tasks such as CAD prediction (Table 7).

## 4. Discussion

This study evaluated the performance of classification models that integrate demographic, anthropometric, biochemical, and psychosocial variables using ML algorithms to achieve a more accurate, rapid, and personalized diagnosis of CAD. The high prevalence and mortality rates associated with CAD highlight the critical need for early diagnosis and risk prediction methods, both at individual and public control levels. Our findings suggest that models developed through multilayered data integration have the potential to provide more comprehensive insights than those reported in similar studies. The integration of demographic, anthropometric, biochemical, and psychosocial data provides a multidimensional view of each patient’s risk profile, allowing for more personalized CAD prediction compared with models that rely on traditional risk factors alone [12,13]. This multimodal structure supports early diagnosis by capturing subtle variations among metabolic and behavioral variables that may not be evident in conventional clinical assessments [14]. In recent research, combining metabolic indices such as triglyceride–glucose ratio and anthropometric features has significantly improved CAD classification performance [13]. Therefore, the multilayered data integration strategy adopted in this study aligns with current evidence emphasizing the value of comprehensive datasets in supporting early and individualized risk stratification for CAD [12,14]. Notably, k-NN, SVM, and ANN stood out with high accuracy (98.33%, 96.67%, and 95.33%, respectively) and strong AUC values (0.99, 0.95, and 0.98, respectively), underscoring their effectiveness in distinguishing CAD from control cases.

Although subgroup identification within the CAD population was not the primary objective, exploratory analysis revealed heterogeneity in metabolic and anthropometric profiles, suggesting potential phenotypic clusters related to obesity, dyslipidemia, and glycemic status. Such variation indicates that future ML-based analyses incorporating unsupervised clustering may help uncover hidden CAD subtypes. Compared with conventional clinical risk tools such as the Framingham Risk Score or SCORE2, the ML models used in this study demonstrated superior discriminative ability, particularly for patients with borderline or mixed-risk profiles. This supports the clinical relevance of data-driven classification approaches that integrate complex variable interactions beyond traditional regression-based methods.

Previous studies of CAD prediction using ML have shown promising progress in this field. For instance, Zhang et al. (2024) reported that LR achieved an AUROC of 0.981, highlighting the importance of age and TG levels as predictors [12]. Similarly, Mehmood et al. (2024) achieved 99% accuracy using an ensemble voting classifier, and emphasized the superior performance of k-NN, SVM, and LR [41]. Despite utilizing a relatively small dataset (*n* = 300), our study achieved comparable or even higher performance, with the k-NN model reaching an AUC of 0.99 and 98.33%. This success can be attributed to the comprehensive nature of the dataset, including 21 biochemical parameters, anthropometric measures, and psychosocial data, as well as the use of a 10-fold cross-validation. However, the advantages of generalizability in large-scale studies must also be acknowledged [2].

One of the most notable aspects of our study was the integration of all demographic, biochemical, and anthropometric variables into a single model. Although previous reports have noted that the use of only selected feature subsets may limit model performance, our results demonstrate that broader datasets contribute positively to model accuracy [1,42]. In addition, the inclusion of all variables in the model from the application of feature selection allowed the data to be run correctly while preserving the richness of the data, supporting the need for clinical decision support systems to provide multidimensional data [20,43].

Our study revealed a strong association between abdominal obesity indicators (waist and hip circumference), metabolic parameters (glucose, HbA1c, TG, and LDL-C), and CAD (*p* < 0.001). These findings are consistent with those of previous studies examining the relationship between obesity phenotypes and cardiovascular diseases [3]. In particular, the detection of low vitamin B12 levels in the CAD group is a new finding, indicating the potential role of metabolic derangements in the pathogenesis of CAD. This result supports the limited studies in the literature examining the relationship between B12 deficiency and cardiovascular risk [6]. However, the causality and clinical significance of this relationship requires further investigation.

Regarding thyroid function, FT4 levels were significantly lower in the CAD group compared with the control group (*p* < 0.001, Table 4). This finding suggests that subtle thyroid hormone variations, even within the FT3 and TSH normal range, may influence cardiovascular risk. Previous studies have reported associations between subclinical thyroid dysfunction and coronary heart disease [44,45], supporting the possible contribution of minor thyroid hormonal changes to CAD pathophysiology. However, the exclusion of patients with overt thyroid disease and the limited sample size warrant cautious interpretation

In addition, the relationship between psychosocial eating behaviors (emotional eating and eating awareness) and the risk of CAD constitutes an innovative aspect of our study. Although emotional eating (mean: 4.01 ± 0.49) and eating awareness (mean: 3.67 ± 0.21) data indicate behavioral factors that may affect the metabolic profiles of individuals with CAD, the contribution of these data to classification performance was limited. This may be explained by the subjective nature of the psychosocial scales and the noise added to the model by the individual response differences. Behavioral factors such as emotional eating have been reported to be associated with obesity and cardiometabolic risk [3], but the integration of these factors into ML models has not yet been sufficiently investigated.

Specifically, the mean Emotional Eating Scale (EES) and Mindful Eating Questionnaire (MEQ) scores were 19.3 ± 5.4 and 24.7 ± 6.1 in the CAD group, and 15.2 ± 5.1 and 29.8 ± 5.9 in the control group, respectively. A total of 12 biochemical parameters (including glucose, HbA1c, LDL-C, HDL-C, triglycerides, creatinine, and uric acid) were integrated alongside anthropometric and psychosocial variables to create the model’s input feature set. While the addition of behavioral data introduced minor noise—as reflected by slightly higher variance in cross-validation accuracy—it improved the model’s interpretability by capturing behavioral dimensions of CAD risk [14]. These results are in line with studies indicating that stress-related or emotional eating patterns may exacerbate metabolic dysregulation and elevate cardiovascular risk [13,14]

Our results showed that utilizing all features without selection contributed to maintaining the data diversity and enhancing the model performance. The richness of the data facilitated accurate discrimination between control individuals and those with CAD. This suggests that fully exploiting information within clinical datasets, rather than narrowing the scope through feature selection, can maximize the potential of decision support systems [19,42]. Furthermore, our findings highlight the critical importance of multidisciplinary data integration in diagnosing complex diseases such as CAD, aligning with previous calls for comprehensive data preprocessing and holistic data utilization strategies [20,46].

The results of our study are remarkable in terms of revealing the superior performance of the k-NN model, especially when compared with the metrics of the results of previous reports. For example, although the results obtained by Abdar et al. and Akella et al. showed that SVM methods can achieve high accuracy rates [1,42], in our study, it was observed that the k-NN model had a much higher overall accuracy and AUC values [19,43]. In addition, similarities were observed with the reported performances of other applications (LR, SVM, ANN, NB, and DT); however, the margin intervals of the NB and DT models on the patient class were parallel to the findings in the previous literature [1,20].

While the k-NN and SVM models achieved high sensitivity (TPR > 96%), the analysis of failure cases—particularly false negatives—is critical for clinical safety. The few missed CAD cases (e.g., three patients in k-NN) likely represent individuals with *atypical or borderline phenotypes*, such as those exhibiting mild dyslipidemia, near-normal glucose levels, or less pronounced abdominal obesity. These patients may fall outside the dominant feature patterns captured by the models [13,14].

Furthermore, the clinical interpretability of these *black-box* algorithms remains a significant challenge. Although our models consistently identified glucose, LDL-C, and waist circumference as key predictors, explaining the rationale behind an individual-level classification remains difficult in non-linear models such as ANN or SVM. To enhance transparency and clinical applicability, future work should incorporate explainable AI (XAI) frameworks—such as SHAP (SHapley Additive exPlanations) or LIME (Local Interpretable Model-Agnostic Explanations)—to visualize feature contributions and improve clinician trust in ML-based decision support systems [12,20].

The contribution of psychosocial data to classification performance was limited. This can be explained by the fact that psychosocial scales are based on subjective reports, and individual response differences may create noise in the data input to the model. However, behavioral variables such as emotional eating have been reported to be associated with obesity and thus cardiometabolic risk [3]; therefore, the potential contributions of these variables should be re-evaluated in further studies with more sophisticated analyses. Beyond emotional and mindful eating, future research should consider incorporating broader eating patterns as well as psychological factors such as anxiety and depression, which may further influence cardiometabolic risk and improve behavioral component modeling.

The NB and DT algorithms used in our study showed lower performance in terms of sensitivity for the patient class. This finding can be attributed to the fact that these algorithms are generally less flexible and have lower adaptability to complex and high-dimensional datasets. Jose et al. (2024) reported that the DT model exhibited lower AUC values compared to LR, and its classification performance was limited in the patient subgroup [47].

When evaluated in terms of model performance comparison, another remarkable finding of our study was that high accuracy levels were maintained despite including all data in the model without feature selection. However, because this approach may pave the way for overfitting by increasing the model complexity, the integration of feature selection techniques such as the Boruta algorithm or SHAP analysis may increase the generalizability of the model [20] as frequently suggested in the literature [12].

Traditional clinical risk scores—such as the Framingham Risk Score [48], SCORE2 [49], or ASCVD Risk Estimator [50]—are widely used for cardiovascular risk stratification but primarily rely on a limited number of demographic and biochemical variables. In contrast, machine learning (ML) models can simultaneously analyze multidimensional and non-linear relationships among a broader set of features, including psychosocial and behavioral parameters, without requiring pre-assigned weighting. Unlike conventional scores, ML algorithms dynamically identify complex patterns that may not be apparent in regression-based methods, thereby offering more personalized and potentially more accurate predictions for CAD. Future research should directly compare ML models with established risk scoring systems to assess incremental predictive value and clinical applicability.

One of the secondary aims of this study was to explore the interplay between eating behavior, metabolic regulation, and CAD risk. Although the psychosocial variables (emotional and mindful eating) showed limited direct contribution to classification performance, their inclusion provided insight into behavioral mechanisms potentially influencing metabolic outcomes. Both scales have been validated in the Turkish population [51]. Emotional eating is known to be associated with stress-induced hyperphagia, insulin resistance, and central adiposity, whereas mindful eating is linked to improved self-regulation and metabolic control [52]. This integration reflects an exploratory attempt to bridge behavioral and physiological dimensions within a data-driven CAD prediction framework.

### Limitations

This study was single-center and had a limited sample size, which limits the generalizability of the findings. Inclusion of all variables in the model without feature selection may have increased model complexity and caused some variables to be included in the model unnecessarily. In addition, only classical supervised algorithms were used, and the ensemble and deep learning methods were excluded.

The contribution of psychosocial data to the classification was exploratory but limited and was not analyzed separately. These limitations can be overcome by developing models with larger and more balanced samples in independent cohorts in the future. Furthermore, other demographic aspects, such as ethnicity and race, were not considered in this study and may have an important influence on CAD risk. Future studies including more diverse populations are warranted to further enhance the generalizability of the findings. Finally, because this study was retrospective in design, a direct comparison between machine learning predictions and cardiologist assessments could not be performed. Future prospective investigations should aim to assess the concordance between model predictions and expert clinical judgments to further validate the diagnostic reliability of ML-based approaches.

## 5. Conclusions

This study evaluated the performance of ML-based classification models integrating demographic, anthropometric, biochemical, and psychosocial variables for the prediction of CAD. Among the six supervised learning algorithms tested, k-NN, SVM, and ANN demonstrated the most robust classification performance, achieving accuracies of 98.33%, 96.67%, and 95.33%, respectively, along with high AUC values of 0.99, 0.95, and 0.98. These findings highlight the effectiveness of data-driven approaches and emphasize their potential clinical applicability in personalized CAD prediction.

These strong model performances can be partly explained by the inclusion of comprehensive metabolic and anthropometric variables, which captured subtle but clinically relevant differences between CAD and non-CAD individuals. Metabolic indicators, such as elevated glucose, HbA1c, LDL-C, TG, and abdominal obesity markers (waist and hip circumference), as well as reduced vitamin B12 levels, were significantly associated with CAD presence (*p* < 0.001). These results align with recent evidence identifying glucose, triglyceride, and TyG index values as key metabolic predictors of CAD [12,13], reinforcing the central role of metabolic dysregulation in disease pathogenesis.

Beyond metabolic predictors, behavioral and psychosocial dimensions also contributed to understanding CAD risk. Incorporating emotional eating and mindful eating variables introduced a novel behavioral layer to the model. Although their statistical contribution to classification was limited—likely due to the subjective nature of self-reported data—their inclusion provided a more holistic representation of patient risk profiles by linking behavioral tendencies with metabolic outcomes [13,14]. Integrating these diverse data domains within a single analytical framework underscores the growing importance of multidimensional modeling in precision cardiology.

While integrating diverse features improved model interpretability, some algorithms—particularly Naïve Bayes and Decision Tree—showed lower sensitivities for the CAD class. This limitation likely stems from their restricted ability to capture complex nonlinear relationships and interdependent variables. Nevertheless, our findings demonstrate that employing a comprehensive feature set without prior feature elimination enhanced overall model robustness and accuracy, emphasizing the benefits of multidomain inclusion for data-driven prediction.

In conclusion, this study demonstrates that ML models, particularly k-NN, SVM, and ANN, can effectively leverage multidimensional data to support early diagnosis and risk assessment of CAD. The integration of metabolic, anthropometric, and behavioral factors offers promising opportunities for developing personalized, data-driven clinical decision support systems aimed at improving cardiovascular health outcomes.

The size of a database and the quality of its data are two effective factors in ML performance. Future research will focus on validating the developed models using larger, multi-center datasets collected in collaboration with hospitals to enhance external validity and reduce potential bias. In addition, upcoming studies will aim to compare the predictive performance of ML-based models with established clinical risk assessment tools such as the Framingham Risk Score, SCORE2, and the ASCVD Risk Estimator. By integrating larger and more diverse datasets, as well as benchmarking against conventional diagnostic pathways, we aim to develop more generalizable, clinically interpretable, and practically applicable models for CAD prediction.

## Figures and Tables

**Table 1:** Summary of machine learning-based studies for coronary artery disease diagnosis.

Authors	Year	Method	Number of Participants	Sampling	Conclusion	Data
Garavand et al. [15]	2022	Logistic Regression, k-NN, Naïve Bayes	Public dataset (~300)	64% train/20% test/16% validation	LR showed strong predictive power using low-cost clinical data	Clean, standardized patient data with no preprocessing
Gudadhe et al. [16]	2020	SVM, Decision Tree, Neural Networks	303 (Cleveland dataset)	80% train/20% test	SVM yielded highest accuracy (>85%)	UCI CAD dataset
Yılmaz & Yağın [17]	2022	RF, LR, SVM	Not specified	10-fold CV	RF achieved highest accuracy (92.9%)	Local clinical dataset
Miao et al. [18]	2016	AdaBoost (ensemble)	UCI datasets (CCF, HIC, LBMC, SUH)	Dataset dependent	SUH dataset showed highest accuracy (96.72%)	Multi-source UCI datasets
Chen et al. [19]	2020	Polynomial SVM	Unknown (CCTA data)	Train/test split	100% accuracy with polynomial-SVM on bifurcation features	Three-dimensional coronary CT angiography
Alizadehsani et al. [20]	2019	Feature selection + ML classifiers	303	10-fold CV	Identified most relevant features with high accuracy	Z-Alizadeh dataset
Mirjalili et al. [13]	2023	SVM with TyG index	2000	Longitudinal cohort	TyG index superior to diabetes marker for CHD prediction	Iranian national cohort
Sammout et al. [21]	2021	Ensemble classifiers	1200+	Stratified CV	Ensemble approach increased generalizability and accuracy	Multi-institutional heart dataset
Sun et al. [22]	2024	Parallel CNN + Autoencoder + SVM	199 (135 CAD, 64 non-CAD)	5-fold CV	Multi-modal (ECG + PCG + coupling) achieved 98.49% accuracy	ECG + PCG + ECG–PCG coupling signals
Saeedbakhsh et al. [23]	2023	SVM, ANN, RF	11,495	Cohort dataset	SVM had highest accuracy (89.73%); ANN also performed well	Isfahan Cohort Study, 19 features
Sayadi et al. [24]	2022	Decision Tree, Deep Learning, LR, RF, SVM, XGBoost	Z-Alizadeh Sani dataset (303)	Pearson feature selection	Only 8 features were effective for CAD diagnosis	Z-Alizadeh Sani dataset
Munmun et al. [14]	2025	LR, RF, SVM	1258	Stratified balanced dataset	RF showed best accuracy (~93.5%)	Combined five heart datasets
Fitriyani et al. [25]	2020	Hybrid SMOTE-ENN and XGBoost	573	270 instances, 13 attributes and 303 instances, 13 attributes respectively	95.90% and 98.40% respectively	Statlog and Cleveland dataset

Abbreviations: CAD, coronary artery disease; ML, machine learning; DT, decision tree; LR, logistic regression; NB, naïve Bayes; SVM, support vector machine; k-NN, k-nearest neighbors; ANN, artificial neural network; CAD, coronary artery disease.

**Table 2:** Demographic Characteristics of the Study Population.

Variable	All Participants (*n* = 300)	Control (*n* = 135)	CAD Present (*n* = 165)	*p*-Value
Age (years)	55.2 ± 7.0	49.4 ± 5.6	58.4 ± 2.7	0.000 *
Sex (Male/Female)	160/140 (53.3%/46.7%)	68/67 (50.4%/49.6%)	92/73 (55.8%/44.2%)	0.341

Abbreviations: CAD, coronary artery disease; *p*, probability value. * Statistically significant (*p* < 0.05).

**Table 3:** Body Composition Measurements.

Variable	All Participants (*n* = 300)	Control (*n* = 135)	CAD (*n* = 165)	*p*-Value
Height (cm)	167.6 ± 9.3	170.1 ± 9.8	161.7 ± 3.5	0.000 *
Weight (kg)	85.0 ± 6.8	78.8 ± 3.7	87.5 ± 6.2	0.000 *
Waist Circumference (cm)	113.3 ± 8.2	109.3 ± 4.2	116.6 ± 9.2	0.000 *
Hip Circumference (cm)	115.8 ± 4.1	114.0 ± 4.3	117.3 ± 3.3	0.000 *
Body Fat Percentage (%)	35.7 ± 6.7	33.7 ± 6.7	40.8 ± 2.7	0.000 *
Muscle Mass (kg)	51.8 ± 9.7	55.4 ± 9.3	43.0 ± 2.9	0.000 *

Abbreviations: CAD, coronary artery disease; *p*, probability value. * Statistically significant difference (*p* < 0.05).

**Table 4:** Laboratory Parameters.

Variable	All Participants (*n* = 300)	Control (*n* = 135)	CAD (*n* = 165)	*p*-Value
HbA1c (%)	6.5 ± 1.0	6.1 ± 0.9	7.4 ± 0.6	0.000 *
Glucose (mg/dL)	114.9 ± 28.0	104.8 ± 26.7	139.3 ± 10.9	0.000 *
TG (mg/dL)	179.3 ± 48.1	164.7 ± 41.9	215.1 ± 43.5	0.000 *
TC (mg/dL)	190.5 ± 23.8	184.3 ± 24.6	205.6 ± 12.6	0.000 *
HDL-C (mg/dL)	47.0 ± 5.0	46.8 ± 5.5	47.5 ± 3.6	0.299
LDL-C (mg/dL)	121.9 ± 21.5	116.1 ± 21.9	135.9 ± 12.1	0.000 *
AST (U/L)	20.45 ± 2.89	21.17 ± 3.67	19.85 ± 1.86	<0.001 *
ALT (U/L)	19.83 ± 5.27	22.76 ± 5.63	17.43 ± 3.44	<0.001 *
ALP (U/L)	66.71 ± 8.17	72.12 ± 7.56	62.28 ± 5.56	<0.001 *
GGT (U/L)	21.66 ± 10.99	25.99 ± 11.37	18.12 ± 9.30	<0.001 *
FT3 (pg/mL)	3.34 ± 0.23	3.37 ± 0.20	3.32 ± 0.25	0.029
FT4 (ng/dL)	1.24 ± 0.09	1.29 ± 0.09	1.19 ± 0.07	<0.001 *
TSH (μIU/mL)	3.1 ± 15.3	3.3 ± 18.2	2.6 ± 0.6	0.665
Ferritin (ng/mL)	42.8 ± 23.1	50.1 ± 23.3	25.0 ± 8.0	0.000 *
Vitamin B12 (pg/mL)	432.3 ± 104.8	518.9 ± 115.1	396.9 ± 76.2	0.000 *
Hgb (g/dL)	14.23 ± 1.22	14.54 ± 1.34	13.97 ± 1.04	<0.001 *
TyG Index	9.29 ± 0.39	9.11 ± 0.41	9.44 ± 0.31	<0.001 *
AIP	0.62 ± 0.16	0.54 ± 0.12	0.69 ± 0.17	<0.001 *
LDL-C/HDL-C Ratio	2.6 ± 0.4	2.5 ± 0.3	2.9 ± 0.2	0.000 *
TC/HDL-C Ratio	4.1 ± 0.4	3.9 ± 0.4	4.3 ± 0.3	0.000 *
Non-HDL-C (mg/dL)	143.4 ± 21.1	137.5 ± 21.3	157.9 ± 11.5	0.000 *

Abbreviations: CAD, coronary artery disease; *p*, probability value; HbA1c, glycated hemoglobin; TG, triglycerides; TC, total cholesterol; HDL-C, high-density lipoprotein cholesterol; LDL-C, low-density lipoprotein cholesterol; AST, aspartate aminotransferase; ALT, alanine aminotransferase; ALP, alkaline phosphatase; GGT, gamma-glutamyl transferase; FT3, free triiodothyronine; FT4, free thyroxine; TSH, thyroid-stimulating hormone; Hgb, hemoglobin; TyG index, triglyceride-glucose index; AIP, atherogenic index of plasma; non-HDL-C, non-high-density lipoprotein cholesterol. * Statistically significant difference (*p* < 0.05).

**Table 5:** Tuned hyperparameters and best values selected using MATLAB Classification Learner App.

Model	Tuned Parameters	Selected Value(s)
Decision Tree (DT)	Maximum number of splits	20
Logistic Regression	Regularization strength	1 (default), Solver: LBFGS
Naive Bayes (NB)	Distribution type	Gaussian
Support Vector Machine (SVM)	Kernel function, Box constraint (C)	RBF Kernel, C = 1
k-Nearest Neighbor	Number of neighbors, Distance metric	k = 5, Euclidean distance
Artificial Neural Network (ANN)	Number of hidden neurons, activation function	1 hidden layer, 10 neurons, ReLU

**Table 6:** Summary of Model Performances.

Model		TPR (%)	FNR (%)	Accuracy (%)
DT [all features]	CAD	96.97	3.03	94.00
	Control	90.37	9.63	
LR [all features]	CAD	90.91	9.09	91.33
	Control	91.85	8.15	
NB [all features]	CAD	94.55	5.45	94.67
	Control	94.81	5.19	
SVM [all features]	CAD	96.97	3.03	96.67
	Control	96.30	3.70	
k-NN [all features]	CAD	98.18	1.82	98.33
	Control	98.52	1.48	
ANN [all features]	CAD	96.97	3.03	95.33
	Control	93.33	6.67	

Abbreviations: DT, decision tree; LR, logistic regression; NB, naïve Bayes; SVM, support vector machine; k-NNs, k-nearest neighbors; ANN, artificial neural network; CAD, coronary artery disease; TPR, true positive rate; FNR, false negative rate.

**Table 7:** Average performance metrics of machine learning models using 10-fold cross-validation.

Model	Accuracy (%)	Precision	Recall	F1 Score	AUC
Decision Tree (DT)	86.00	0.84	0.85	0.84	0.89
Logistic Regression (LR)	91.67	0.90	0.92	0.91	0.95
Naive Bayes (NB)	88.33	0.87	0.88	0.87	0.92
Support Vector Machine (SVM)	96.67	0.96	0.97	0.96	0.98
k-Nearest Neighbor (k-NN)	98.33	0.99	0.98	0.98	0.99
Artificial Neural Network (ANN)	95.00	0.95	0.95	0.95	0.97

## Data Availability

The data are provided in the manuscript. If needed, the datasets generated and/or analyzed during the current study are available from the corresponding author upon reasonable request.

## Abbreviations

AIP	Atherogenic Index of Plasma
ANN	Artificial Neural Network
AUC	Area Under the Curve
BMI	Body Mass Index
CAD	Coronary Artery Disease
CAG	Coronary Angiography
CTA	Computed Tomography Angiography
DT	Decision Tree
ECG	Electrocardiography
Echo	Echocardiography
FNR	False Negative Rate
HbA1c	Glycated Hemoglobin
HDL-C	High-Density Lipoprotein Cholesterol
LDL-C	Low-Density Lipoprotein Cholesterol
LR	Logistic Regression
Non-HDL-C	Non-High-Density Lipoprotein Cholesterol
ML	Machine Learning
NB	Naïve Bayes
ROC	Receiver Operating Characteristic
SVM	Support Vector Machine
TG	Triglyceride
TPR	True Positive Rate
TyG	Triglyceride-Glucose Index
